# A laboratory-scale pretreatment and hydrolysis assay for determination of reactivity in cellulosic biomass feedstocks

**DOI:** 10.1186/1754-6834-6-162

**Published:** 2013-11-14

**Authors:** Edward J Wolfrum, Ryan M Ness, Nicholas J Nagle, Darren J Peterson, Christopher J Scarlata

**Affiliations:** 1 National Bioenergy Center, National Renewable Energy Laboratory, 15013 Denver West Parkway, Golden, CO 80401, USA

## Abstract

**Background:**

The rapid determination of the release of structural sugars from biomass feedstocks is an important enabling technology for the development of cellulosic biofuels. An assay that is used to determine sugar release for large numbers of samples must be robust, rapid, and easy to perform, and must use modest amounts of the samples to be tested.

In this work we present a laboratory-scale combined pretreatment and saccharification assay that can be used as a biomass feedstock screening tool. The assay uses a commercially available automated solvent extraction system for pretreatment followed by a small-scale enzymatic hydrolysis step. The assay allows multiple samples to be screened simultaneously, and uses only ~3 g of biomass per sample. If the composition of the biomass sample is known, the results of the assay can be expressed as reactivity (fraction of structural carbohydrate present in the biomass sample released as monomeric sugars).

**Results:**

We first present pretreatment and enzymatic hydrolysis experiments on a set of representative biomass feedstock samples (corn stover, poplar, sorghum, switchgrass) in order to put the assay in context, and then show the results of the assay applied to approximately 150 different feedstock samples covering 5 different materials. From the compositional analysis data we identify a positive correlation between lignin and structural carbohydrates, and from the reactivity data we identify a negative correlation between both carbohydrate and lignin content and total reactivity. The negative correlation between lignin content and total reactivity suggests that lignin may interfere with sugar release, or that more mature samples (with higher structural sugars) may have more recalcitrant lignin.

**Conclusions:**

The assay presented in this work provides a robust and straightforward method to measure the sugar release after pretreatment and saccharification that can be used as a biomass feedstock screening tool. We demonstrated the utility of the assay by identifying correlations between feedstock composition and reactivity in a population of 150 samples.

## Background

The production of cellulosic biofuels via biochemical pathways typically consists of three discrete processing steps: chemical pretreatment, enzymatic hydrolysis, and microbial conversion [1,2]. The role of the first two steps is to generate soluble carbohydrates, whereas the goal of the final step is the conversion of these carbohydrates to fuels. Although fermentation of these soluble carbohydrates (principally glucose and xylose) to ethanol has been the main focus of research over the last several decades, there has been significant interest more recently in the production of other fuels and chemicals using both biological and chemical processes [3-8].

Regardless of the specific nature of the sugar conversion step, the ability to derive soluble carbohydrates from biomass feedstocks is critical to the overall economics of the biofuel production process [9-11]. Understanding soluble sugar production after pretreatment and enzymatic hydrolysis is thus an important task. A rapid and facile laboratory-scale assay that can differentiate among different feedstocks with respect to reactivity (for example, the amount of soluble carbohydrates produced per unit biomass) can help identify highly reactive or recalcitrant feedstock samples, which could then be investigated in more detail.

The goal of such assays is to estimate the biomass feedstock reactivity at a scale suitable for processing many samples in a short period of time, usually to identify specific samples with unusual behavior compared to most other samples. There are two general approaches to developing a rapid assay: to miniaturize and automate a larger-scale assay using custom-designed laboratory hardware (for example, robotics) [12-15], or to use a more rapid assay based on a secondary analysis technique, such as spectroscopy [16-20]. Such spectroscopic approaches are called secondary techniques because they rely on primary data generated using a laboratory assay to develop calibrations. The goal of this work is to demonstrate a reproducible laboratory-scale feedstock reactivity screening assay based on commercially-available hardware and a standard enzymatic hydrolysis assay. Such an assay does not require custom-designed equipment, and can provide relatively rapid results using trained operators.

In addition to the spectroscopic studies mentioned above, there has been other work in laboratory-scale assays for pretreatment and enzymatic hydrolysis. For example, work by the Consortium for Applied Fundamentals and Innovation (CAFI) comparing a number of different pretreatment technologies presented a number of laboratory assays utilizing different pretreatment chemistries and reactor vessels [21,22]. Other reports have used multiple laboratory-scale pretreatment reactors in the same work [23,24]. There have been a number of studies using the automated solvent extraction approach for laboratory-scale pretreatment assays using hot water [25] and dilute acid [26,27] pretreatment chemistries.

As mentioned above, there have been a number of reports on the use of spectroscopic methods to predict the release of sugars (or the production of ethanol) based on laboratory assays [17-20,28]. This work focuses on the primary assay itself.

This report builds on the existing literature in two ways. First, we investigated the effect of pretreatment temperature on carbohydrate release for four representative feedstocks (corn stover, switchgrass, sorghum, and poplar); this shows the trade-offs associated with the development of a screening assay, which (by definition) is performed with a specific set of conditions. Second, we present the results of the screening assay applied to approximately 150 different herbaceous feedstock samples and discuss these results in detail, including the identification of trends in the data associated with biomass feedstock composition.

## Results and discussion

### A note on nomenclature and calculations

For any assay there is typically more than a single way to express the results of the assay. In this work we report the results in terms of sugar release and sugar yield. We define sugar release as the mass of glucose and/or xylose (the sum of monomeric and oligomeric) released per unit mass of biomass starting material. We define xylan yield as the fraction of xylan released as xylose, including the anhydro correction factor; the ratio of the hydrolyzed sugar monomer to the anhydrous structural repeat unit. We define the glucan yield similarly, except we also include contributions from non-structural glucose (from sucrose) and starch. Thus, both the glucan and xylan yield calculation require knowledge of the composition of the starting material. We define reactivity as the total yield of glucose and xylose from the total structural and nonstructural carbohydrates.

For pretreatment experiments, the concept of reactor severity [24,26,29,30] or severity factor is commonly used to characterize the combination of time and temperature in a given reactor. Severity is a nonlinear combination of reactor temperature and time. We did not use severity in this work. For all experiments the heating and static times in the ASE 350 reactor were held constant (7 minutes and 6 minutes, respectively), while the cell temperature varied. Although the severity factor could be calculated a number of ways (the static time alone, sum of the heating and static times, the sum of the fraction of the heating time above a certain temperature and the static time), the results would be the same for all screening data presented in this work. Because the calculation of severity explicitly excludes any consideration of the ratio of acid to biomass, it is unlikely that other reactor geometries operating at the same severities used in this work (for example, a flow-through reactor with near-instantaneous heating or a microwave system), or even the same reactor system operated in a different flow mode would provide identical conversion data. We believe the concept of severity is most useful for interpreting data in a single-reactor geometry and operating mode.

All release, yield, and reactivity calculations are included as Additional file 1. As mentioned above, the primary results of the enzymatic hydrolysis assay are reported as the mass of sugar released per unit mass of biomass added. The mass of sugar released is calculated as the product of a concentration measurement and a volume measurement. This calculation is an approximation; we assume the liquid volume of the system to be 10 mL, which is an overestimation of the actual liquid volume, as some solid residue remains in the flask at the end of enzymatic hydrolysis. To determine the results of enzymatic hydrolysis for larger-scale systems, we would perform fraction insoluble solids (FIS) measurement [31] to determine the liquid volume exactly. However, the small amount of material used for this assay, and the need for a high-throughput assay itself, makes the FIS measurement infeasible at this scale. Since the actual liquid volume is less than our estimate of 10 mL (as for example, lignin will remain a solid at the end of the experiment) we recognize that the enzymatic hydrolysis assay slightly overestimates the sugar release values.

### Accuracy and precision of assay

**Figure 1:**
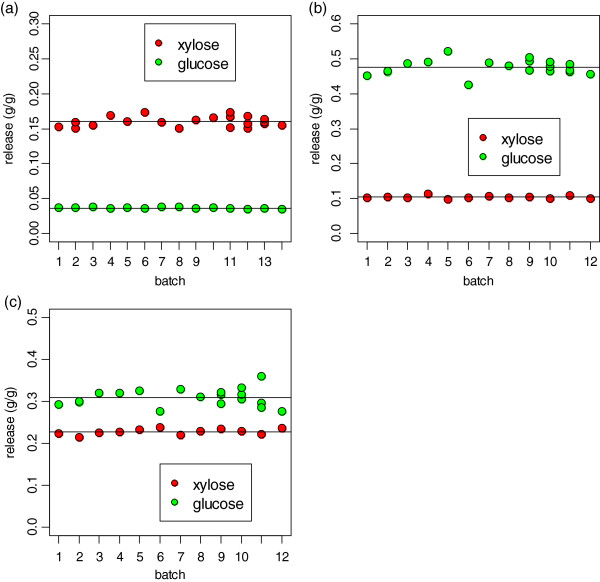
**Results of method control experiments using corn stover control material.** Measured glucose and xylose release from **(a)** pretreatment assay, **(b)** enzymatic hydrolysis assay, and **(c)** combined pretreatment (PT) + enzymatic hydrolysis (EH) assay. As expected, the majority of xylose was released during pretreatment whereas the majority of glucose was released during enzymatic hydrolysis. The variability of the glucose release (SD = 2.1%) was greater than the variability of the xylose release (SD = 0.7%). The variability of these replicates limits the discriminating power of the assay.

A well-characterized corn stover material was used as an internal method validation sample for both the pretreatment and enzymatic hydrolysis assays. Including replicates, this sample was assayed 21 times in 14 separate pretreatment batches and 12 subsequent enzymatic hydrolysis batches (the batch size for enzymatic hydrolysis was slightly larger than for pretreatment). We can use these repeated data to measure the variability of the method. The data in Figure 1 show the glucose and xylose release from repeated assays of this material. Figure 1a and b show the results of the pretreatment and enzymatic assay separately, and Figure 1c shows the results of the combined assay. The xylose release was higher than glucose release during pretreatment and the opposite was true for enzymatic hydrolysis, completely expected given the purpose and biochemistry of the two assays. The mean and standard deviations of the key results from these replicated assays are shown in Table 1. For the overall assay, the mean xylose release from the corn stover control was 0.228 ± 0.007 g/g and the mean glucose release from the corn stover control was 0.309 ± 0.021 g/g. The overall assay has a higher uncertainty for glucose release than for xylose release because the enzymatic assay, in which most of the glucose is released, has a higher variability than the pretreatment assay. Regardless of the source(s) of the variability in these measurements, they limit the ability of the combined assay to identify differences between and among different sample types. We discuss this issue in detail below.

**Table 1:** Mean and standard deviations of replicate measures of xylose release from pretreatment, glucose release from enzymatic hydrolysis, and overall xylose and glucose release from the combined pretreatment and enzymatic hydrolysis assay for the corn stover control sample used for each assay batch, and the pooled standard deviation for all replicated samples

	**Xylose release (PT)**		**Glucose release (EH)**		**Xylose release (PT + EH)**		**Glucose release (PT + EH)**	
	**Mean**	**SD**	**Mean**	**SD**	**Mean**	**SD**	**Mean**	**SD**
Corn stover control samples	0.160	0.007	0.476	0.022	0.228	0.007	0.309	0.021
All replicated samples	--	0.009	--	0.014	--	0.009	--	0.011

The mean of all replicated samples is meaningless in this context, and is not shown. PT, pretreatment; EH, enzymatic hydrolysis.

### The pretreatment/enzymatic hydrolysis assays as variables

The results reported here are for dilute acid pretreatment followed by enzymatic hydrolysis. As mentioned previously, other pretreatment chemistries are widely used (for example, steam explosion, ammonia, hot water, and ionic liquid), and different enzyme formulations are typically used for different pretreatment chemistries. For example, Kumar and Wyman [32] observed that xylanase supplementation during enzymatic hydrolysis was more beneficial for some pretreatments than for others. This is not a limitation of the pretreatment process, but rather a recognition that pretreatment chemistries differ; the greater the amount of xylan removed during pretreatment, the less helpful additional xylanase supplementation during enzymatic hydrolysis. Similarly, enzymatic hydrolysis enzymes are subject to inhibition and deactivation by compounds produced during pretreatment [33].

For this work, we used a single pretreatment chemistry (dilute sulfuric acid) and a single enzyme formulation applied at a high mass loading in order to focus on the effect of one major pretreatment variable (temperature) on observed sugar yields. The reader is reminded that the selection of the pretreatment chemistry, other pretreatment variables (for example, acid-biomass ratio, reaction time, biomass particle size), the enzyme formulation, loading, and conditions used for enzymatic hydrolysis are in fact variables that could be investigated separately. Such an extensive study is outside the scope of this work.

### Variable temperature pretreatment/enzymatic hydrolysis experiments

**Figure 2:**
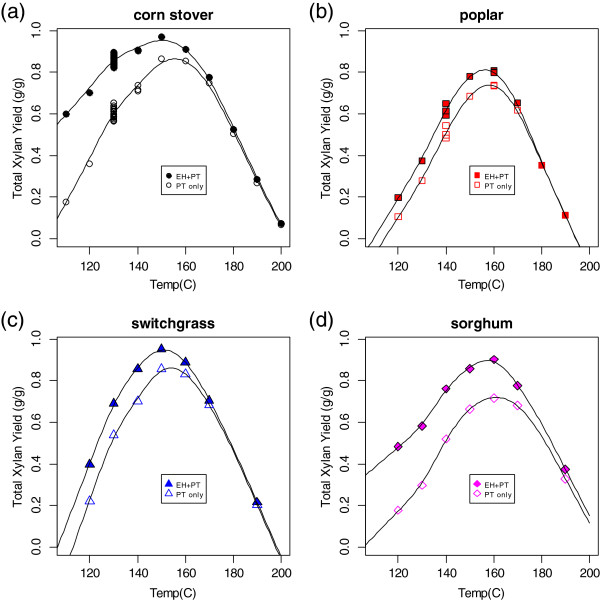
**Total (sum of monomeric and oligomeric) xylan yield (fraction of xylan originally present in biomass feedstock released as xylose) from dilute acid pretreatment alone (hollow symbols) and pretreatment followed by enzymatic hydrolysis (filled symbols) as a function of pretreatment temperature. (a)** Corn stover **(b)**; poplar; **(c)** switchgrass; **(d)** biomass sorghum. All pretreatment experiments were performed with 3 g (dry weight) biomass, 30 mL of 1% sulfuric acid with a 7-minute heating time and a 6-minute static time in a 66-mL zirconium cell. All enzymatic hydrolysis experiments were performed at 10% solids using an enzyme loading of 40 mg/g biomass. For all feedstock types the maximum xylose yield occurs at a temperature of 150°C or 160 C, respectively. However, the maximum difference between the highest and lowest maximum yield values (corn stover and poplar) occurs at a reactor temperature of 130°C.

**Figure 3:**
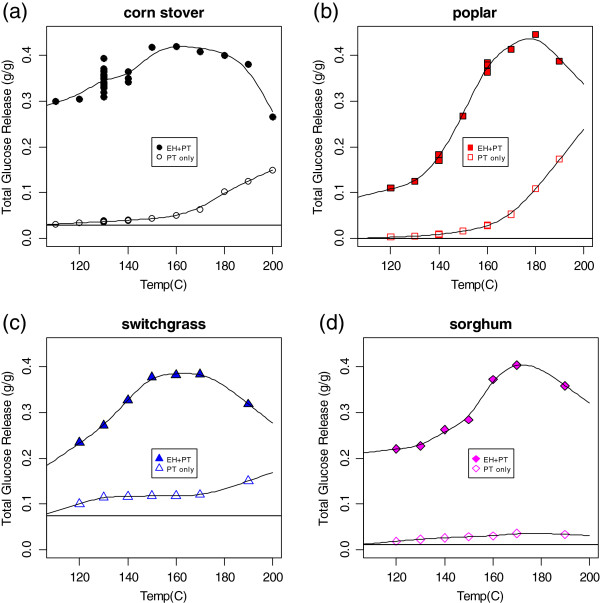
**Total (sum of monomeric and oligomeric) glucose release (fraction of biomass feedstock sample dry weight released as glucose) from dilute acid pretreatment alone (hollow symbols) and pretreatment followed by enzymatic hydrolysis (filled symbols) as a function of pretreatment temperature. (a)** Corn stover; **(b)** poplar; **(c)** switchgrass; **(d)** biomass sorghum. Experimental conditions were the same as in Figure 2. Some glucose was released during pretreatment; the horizontal line in each plot shows the amount of non-structural glucose, derived from starch and sucrose (see text).

**Figure 4:**
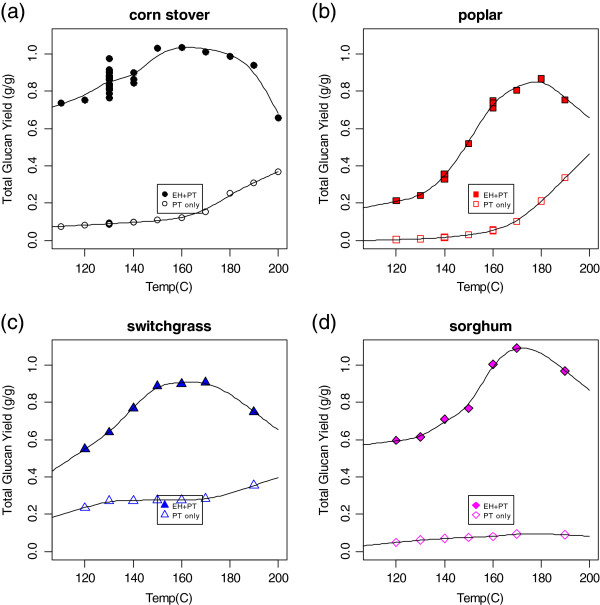
**Total (sum of monomeric and oligomeric) glucan yield (fraction of glucan originally present in biomass feedstock, both structural and nonstructural, released as glucose) from dilute acid pretreatment alone (hollow symbols) and pretreatment followed by enzymatic hydrolysis (filled symbols) as a function of pretreatment severity. (a)** Corn stover; **(b)** poplar; **(c)** switchgrass; **(d)** biomass sorghum. Experimental conditions were the same as in Figure 2. The yield calculations are based on both structural and non-structural contributions (for example, cellulose, starch, sucrose; see text).

**Figure 5:**
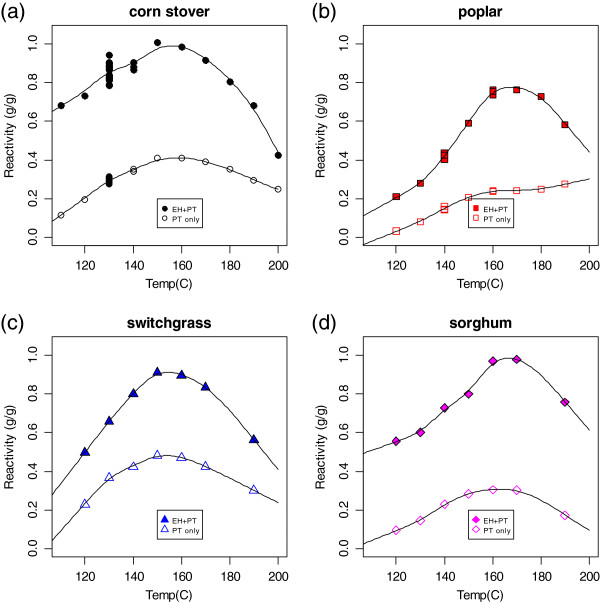
**Total reactivity (fraction of glucan and xylan originally present in biomass feedstock, both structural and nonstructural, released as glucose or xylose) from dilute acid pretreatment alone (hollow symbols) and pretreatment followed by enzymatic hydrolysis (filled symbols) as a function of pretreatment severity. (a)** Corn stover; **(b)** poplar; **(c)** switchgrass; **(d)** biomass sorghum. Experimental conditions were the same as in Figure 2. The yield calculations are based on both structural and non-structural contributions (for example, cellulose, starch, sucrose; see text).

The results of the variable temperature pretreatment experiments are summarized in Figures 2, 3, 4, and 5. The total (monomeric and oligomeric) xylan yields from pretreatment alone and from pretreatment followed by enzymatic hydrolysis for four different biomass feedstocks (corn stover (a), poplar (b), switchgrass (c), and sorghum (d)) across a range of pretreatment temperatures are shown in Figure 2. The smooth curves through the data are cubic splines added to guide the eye of the reader; they do not represent a theoretical model. There are a large number of points at 130°C for the corn stover plot (Figure 2a). These are the method controls, shown as a time series in Figure 1.

For all feedstocks, the xylan yield values showed a maximum at a reaction temperature of 150°C or 160°C. Higher reaction temperatures resulted not only in a reduction of xylose release, but in the formation of furfural, indicating nonproductive conversion of the xylose (data not shown). There was variation in the maximum total xylose yield among the four feedstock types, with corn stover and switchgrass having yields over 90% and sorghum and poplar having yields closer to 80%. For pretreatment temperatures below approximately 170°C, enzymatic hydrolysis releases additional xylose. The amount of additional release was greatest at the lowest pretreatment temperature, although the difference was less pronounced for switchgrass and poplar.

The total (monomeric and oligomeric) glucose release from pretreatment alone and from pretreatment followed by enzymatic hydrolysis as a function of pretreatment temperature for the four different feedstocks are shown in Figure 3. As expected, most of the glucose was released during enzymatic hydrolysis. The general trends for glucose release in this figure are similar to the trends for the xylose yield data in Figure 2, except the maximum glucose release occurred at a pretreatment condition of either 160°C or 170°C. As the pretreatment temperature increased, the amount of glucose released from the pretreatment step alone increased. Note also the repeated data points at 130°C for the corn stover plot (Figure 3a), showing higher variability than the corresponding xylose release data. The horizontal line in each plot in Figure 3 is the amount of nonstructural glucose present in the materials, the sum of the glucose contributions from soluble sucrose and starch (after anhydro correction). Note that in all cases, the glucose release during pretreatment exceeded this value, suggesting that some structural glucan was released during pretreatment, likely from hemicellulose depolymerization.

Figure 4 shows the glucan yield from pretreatment alone and from pretreatment followed by enzymatic hydrolysis as a function of pretreatment temperature for the same four feedstocks. Again, the yield included structural and nonstructural glucose sources. These glucose yield data represent the overall yields from both pretreatment and enzymatic hydrolysis, but must be viewed with caution. First, as discussed previously, the enzymatic hydrolysis assay overestimates the glucose yield; it is unlikely that we have achieved almost 100% glucose yield from the corn stover and over 100% glucose yield from sorghum. Note also that the yield calculations require knowledge of the composition of the feedstocks; these data are unlikely to be available as part of large-scale feedstock screening because of the time-intensive nature of biomass compositional analysis.

The combined yield of xylan and glucan is commonly referred to as reactivity. The reactivity of the four feedstocks as a function of pretreatment temperature is shown in Figure 5.

### Optimal pretreatment conditions for screening

The goal of a high-throughput screening assay is to discriminate among different feedstock samples based on one or more criteria. For this work, we sought an assay that would provide information about the relative merit of different feedstocks with respect to cellulosic sugar production, as measured by the sugar release and yield after pretreatment and enzymatic hydrolysis.

**Figure 6:**
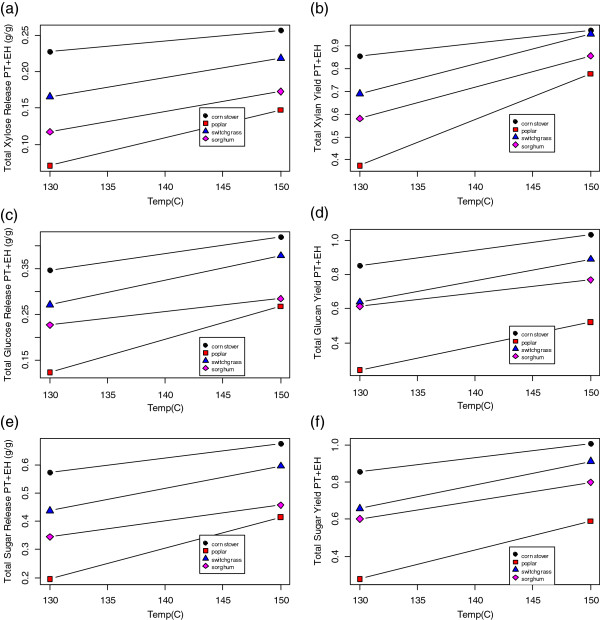
**Effect of pretreatment temperature on results from combined pretreatment-enzymatic hydrolysis assay for four representative feedstocks. (a)** Total xylose release; **(b)** total xylan yield; **(c)** total glucose release; **(d)** total glucan yield, **(e)** toal sugar release, **(f)** toal sugar yield. Larger differences in the experimental results among the four representative feedstocks are apparent at a pretreatment temperature of 130°C than at a pretreatment temperature of 150°C.

The generation of a complete reactivity versus pretreatment temperature profile, such as shown in Figure 5 for each sample to be screened, would multiply any screening effort by an order of magnitude compared to a single-point screening, and varying the enzymatic hydrolysis conditions (for example, enzyme loading) would add another order of magnitude. It seems necessary to pick a single pretreatment and enzymatic hydrolysis condition for screening. How is this condition to be selected, and what should be measured? In Figure 6 we compare the results of the combined assay for the four feedstocks for (a) xylose release, (b) xylan yield, (c) glucose release, (d) glucan yield, (e) total sugar release, and (f) total sugar yield (reactivity) at 130°C and 150°C. These data show that differences among the four model feedstocks depend in part on which of these six values is used to discriminate, and at what temperature the pretreatment portion of the assay is performed.

From the xylose release data in Figure 6a, we note that xylose release was higher for all four feedstocks at the higher temperature, but that a relative ranking of the four feedstocks for xylose release would be the same at either temperature. The lower pretreatment temperature of 130°C provides the largest differences among the four feedstocks for xylose release, but the higher pretreatment temperature of 150°C provides a higher release, more typical for a biomass conversion process. Thus, if the measured assay value is the xylose release, running the assay at either temperature provides the same sample ranking, and the lower temperature may provide better discrimination among samples.

When the measured assay value was the xylan yield (Figure 6b), the ranking of the samples at 130°C was the same, but at 150°C, the ranking results were slightly different; the samples with the two highest xylose releases now had essentially equivalent xylan yields. It would be much more difficult to identify a statistically significant difference between corn stover and switchgrass at the higher temperature based on xylan yield.

When the measured assay value was the glucose release (Figure 6c) we saw the same relative ranking at the lower temperature (130°C) as in Figure 6a and Figure 6b. However, at the higher temperature (150°C), the glucose release from the poplar and switchgrass were equivalent. When the measured assay value was the glucan yield (Figure 6d) we saw slightly different behavior than for glucose release. At both temperatures, it was difficult to distinguish between the sorghum and switchgrass samples, while the glucan yields from corn stover and poplar were distinct. The total sugar-release data in Figure 6e show similar discriminating results as in Figure 6c, and the reactivity data in Figure 6f show similar results as in Figure 6d. As glucose is the major sugar in these feedstocks, similar results were expected when the metric was based on glucose and then on total sugar (glucose plus xylose).

In general, the data in Figure 6 show that the higher pretreatment temperature (150°C) increased the sugar release and yield data to values more representative of actual process conditions, but also reduced the spread in these data, making discrimination among samples more difficult. We chose to perform the screening experiments using a pretreatment temperature of 130°C, because differences among the four model feedstock samples were greater at this temperature than at 150°C. It could be reasonably argued that a pretreatment temperature of 150°C would have been a better choice for exactly the same reason; differences among feedstock samples would have been harder to identify, making any identified differences more likely to translate to real differences in a full-scale process. However, our goal for the screening experiments was to attempt to identify significant differences in reactivity among different feedstock types, so we believe the lower temperature pretreatment assay was a better choice for this particular screening experiment.

### Screening experiments

**Figure 7:**
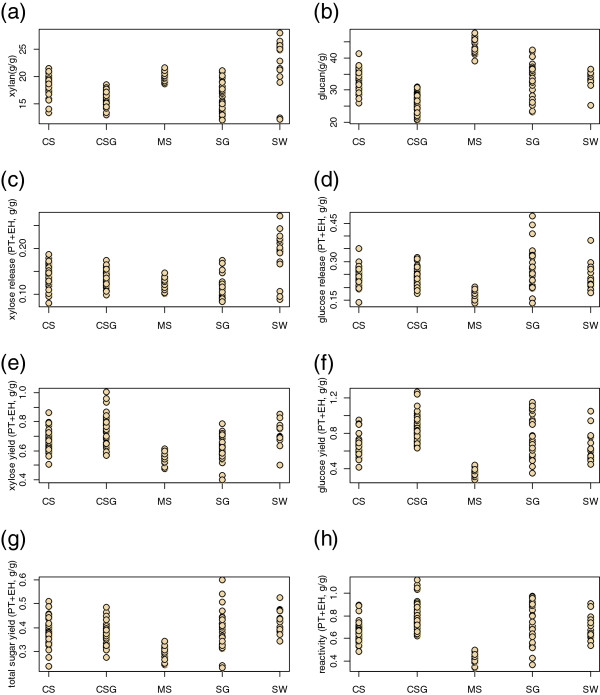
**Results from combined pretreatment-enzymatic hydrolysis assay for 156 feedstock samples. (a)** Xylan content; **(b)** glucan content; **(c)** total xylose release; **(d)** total glucose release; **(e)** total xylan yield; **(f)** total glucan yield; **(g)** total sugar (glucose + xylose) release; **(h)** reactivity. Samples are grouped by feedstock type: CS, corn stover; CSG, cool season grass; MS, miscanthus; SG, sorghum; SW, switchgrass. There was much variation in structural carbohydrate content and all six measures of reactivity within the feedstock types.

The results of the compositional analysis and screening experiments are shown in Figure 7, with the results separated by the five species tested: corn stover (CS), cool season grasses (CSG), miscanthus (MS), sorghum (SG), and switchgrass (SW). Plots (a) and (b) show the xylan and glucan content of the samples, plots (c) and (d) show the xylose and glucose release, plots (e) and (f) show the xylan and glucan yield, and plots (g) and (h) show the total sugar release and reactivity.

Comparing the compositional analysis results in Figure 7, we see the corn stover, sorghum, and switchgrass samples had larger variability in xylan content compared to the cool season grasses and miscanthus samples. Differences in the population means for xylan content were not statistically significant (Tukey honestly significant difference (HSD) test, *P* = 0.05) for the cool season grasses and either corn stover and sorghum (CSG-CS, CSG-SG), and for corn stover and miscanthus (CS-MS); all other between-species differences were statistically significant. Similar results are apparent for the glucan data, with the sorghum and corn stover samples being most variable, and the cool season grasses and miscanthus samples being least variable. The miscanthus samples were highest in glucan, and the cool season grasses the lowest (Tukey HSD, *P* = 0.05). The population means for the glucan content of sorghum, switchgrass, and corn stover were not statistically significantly different (Tukey HSD, *P* = 0.05); all other species differences were statistically significant.

The xylose and glucose release and yield, as well as the total sugar release and reactivity data showed the miscanthus samples to be less variable than all the other species. The yield data were generally (but not uniformly) more variable than the release data. The cool season grasses had the highest glucan yields and the miscanthus samples the lowest (*P* = 0.05); other inter-species differences in total sugar release and reactivity are less clear.

The generalizations and conclusions from the data (shown in Figure 7) discussed above are with respect to the populations we studied. While the miscanthus samples in this study were higher in glucan than the cool season grasses in this study, we do not conclude that this is generally true about miscanthus and cool season grasses. We do not have a detailed understanding of the genotypic or agronomic history of these samples (for example, germplasm, growing conditions). A rapid screening method like the one presented here is most useful when combined with detailed agronomic history of the samples being screened.

**Figure 8:**
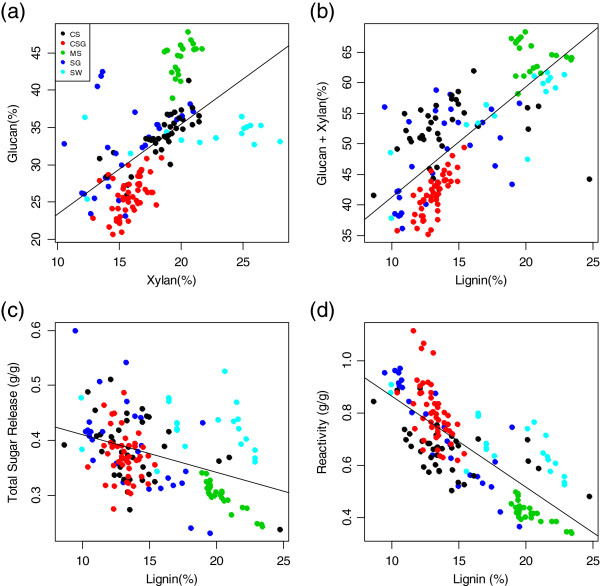
**Correlation of compositional and assay results. (a)** Glucan content versus xylan content; **(b)** sum of glucan and xylan content versus lignin content; **(c)** total sugar release versus lignin content; **(d)** reactivity versus lignin content. Samples are colored by feedstock type: CS, corn stover; CSG, cool season grass; MS, miscanthus; SG, sorghum; SW, switchgrass. Increasing lignin content was correlated with increasing xylan and glucan content, but also with decreasing total sugar release and reactivity (see text).

Looking at the data in aggregate provides further insight regarding these samples. In Figure 8a we see that the glucan and xylan content of the samples were generally correlated. The miscanthus (MS) samples showed the least compositionally variability, while the corn stover (CS) samples showed the greatest variability in glucan content and the switchgrass samples showed the greatest variability in xylan content. The lignin content of the samples was generally correlated with the sum of glucan and xylan content (Figure 8b). The miscanthus samples were highest in lignin while the cool season grass samples were lowest. The effect of lignin content on total sugar release and reactivity are shown in Figure 8c and d. As the lignin content increased the total sugar release and reactivity decreased. Clearly lignin content was positively correlated with glucan and xylan content, but negatively correlated with total sugar release and yield. All of these correlations were statistically significant (*P* = 0.05). The positive correlation with the structural sugars was due to the increase in total structural materials and the corresponding decrease in extractives. The negative correlation between lignin and total sugar release and reactivity suggests lignin may interfere with sugar release during the assay, or that more mature samples (with higher structural sugars) may have more recalcitrant lignin. This is consistent with results seen for poplar [34] but not with results seen for miscanthus [35]. Since increased lignin content was strongly correlated with increased structural carbohydrates, the decreased yields at higher structural carbohydrate content may be related to product inhibition during enzymatic hydrolysis [35]. Regardless of the cause of this phenomenon, we were able to identify it only because of the availability of the aggregate data, supporting the utility of a high-throughput pretreatment/enzymatic hydrolysis assay.

We performed triplicate experiments on approximately 10% of the 155 samples, and the standard deviation of these replicates (pooled by feedstock type) is shown in Table 1. The glucose release and yield data show approximately the same variability as the corn stover control samples discussed earlier, suggesting that we executed the combined assay consistently throughout the course of the work. The use of replicated samples for a high-throughput assay provides another measure of the precision of the assay over time.

However, the combined pretreatment and enzymatic hydrolysis assay presented in this work does provide precise and robust data on sugar release for cellulosic biomass feedstocks. With careful selection of experimental conditions in each of the two assays, and with a good understanding of the agronomic backgrounds (pedigree) of the samples, the assay can be a powerful tool to investigate differences in cellulosic feedstock samples.

As mentioned previously, there have been a number of reports on the use of secondary spectroscopic methods to predict the release of sugars. Although this work reports only the results of the primary assay itself, we have successfully built near-infrared (NIR) calibration models for both composition and reactivity for the samples presented in this work; we will report on this assay in the near future.

## Conclusions

In this work we have presented a method to perform a two-step pretreatment-saccharification assay to measure the sugar release (mass sugar released per unit of oven-dry mass of biomass) of biomass samples. The assay uses a commercially available automated solvent extraction system for pretreatment followed by a small-scale enzymatic hydrolysis step. The assay allows multiple samples to be screened simultaneously, and uses only approximately 3 g of biomass per sample. If the composition of the biomass sample is known, the results of the assay can be expressed as sugar yield (fraction of structural carbohydrate present in the biomass sample released as monomeric sugars). We first investigated the effect of pretreatment conditions on sugar release and yield for four representative biomass samples, and then screened more than 150 different biomass samples using the technique.

The results of the screening work showed a statistically significant negative correlation between lignin content and total sugar release and reactivity. This result suggests that lignin may interfere with sugar release, or that more mature samples (with higher structural sugars) may have more recalcitrant lignin. Regardless of the cause of this phenomenon, the assay allowed us to identify the correlation in an aggregate of 150 samples, demonstrating that it can be a powerful tool to investigate differences in cellulosic feedstock samples.

## Methods

### Biomass compositional analysis

For both pretreatment and enzymatic hydrolysis experiments, all feedstock composition, and hydrolysate liquors organic acid, monomeric sugar, and total sugar concentrations were determined using appropriate National Renewable Energy Laboratory (NREL) laboratory analytical procedures (LAPs) [36-38].

### Biomass feedstock samples

A variety of biomass feedstock samples were used in this work. For the variable pretreatment temperature experiments, four different feedstocks were used: corn stover, poplar, switchgrass, and forage sorghum. For the screening experiments, samples including corn stover, miscanthus, sugar cane bagasse, sorghum, switchgrass, and cool season perennial grasses were used. All biomass samples were dried to less than 10% moisture and milled using a knife mill (Thomas Scientific, Swedesboro, NJ, USA) to pass through a 2-mm screen. The samples were not sieved after milling [39].

### Pretreatment assay

Pretreatment experiments were performed using an ASE 350 accelerated solvent extractor (Dionex, Sunnyvale, CA, USA). For all pretreatment experiments in this work, the ASE 350 was operated in fixed-volume mode, which had less variability than previous modes we had used (data not shown). All experiments were performed using 3.0 ± 0.02 g biomass and 30 mL of 1% sulfuric acid, resulting in a solids loading of approximately 10% and an acid-to-biomass loading of 0.08 g/g. The temperature of the reaction vessels was varied between 110°C and 200°C for the variable temperature experiments, and held at 130°C for the screening experiments. Zirconium cells with a volume of 66 mL were used as reaction vessels and pretreatment hydrolysate liquors were collected in 250-mL glass bottles.

For each experiment the cell was filled with 3.0 ± 0.02 g biomass. The cell was then filled with 30 mL of acid and brought to the reaction temperature. Each experiment consisted of a 7-minute heating period followed by a 6-minute static time. Following pretreatment, the cell temperature was reduced to 100°C and 100 mL of de-ionized water was introduced to the cell. This rinsate was collected in the same 250-mL bottle as the pretreatment liquor. The rinse volume was sufficient to remove essentially all liquor from the solids; sugar concentration measurements in the residual liquor from the pretreated solids were typically below detection. A total of fourteen batches of nineteen samples were run, with at least one sample run in triplicate per batch, and one sample repeated between batches. An aliquot of corn stover was used as a method validation sample (MVS) for every batch.

Following the pretreatment and rinse steps, the 250-mL collection bottles were weighed to determine the mass of fluid collected and the rinsate volume was quantitatively transferred to a 200-mL volumetric flask and brought to volume with deionized water. A 15-mL aliquot was removed from the normalized volume and filtered for determination of total and monomeric sugars and organic acids. A pH measurement was made on the normalized volume.

Each extraction cell was weighed following pretreatment. The mass of washed pretreated solids was determined and the washed pretreated biomass was transferred to a 50-mL plastic Falcon tube. The solids content of an aliquot of the washed solids was determined using an infrared moisture balance. The washed solids and liquors were stored in a refrigerator until the liquors underwent compositional analysis or the solids underwent enzymatic hydrolysis. In several preliminary experiments, we determined a complete mass balance around the pretreatment process and obtained mass closures of 100 ±3% (data not shown).

### Enzymatic hydrolysis assay

The enzymatic hydrolysis assay was substantially similar to the NREL LAP, *Enzymatic Hydrolysis of Lignocellulosic Biomass*[40]. A total of 12 separate batch experiments of 20 to 40 samples were performed. For each experiment, a subsample of the washed solids from the pretreatment experiment was added to a 25-mL Erlenmeyer flask, and appropriate amounts of citrate buffer and enzyme were added to make a 10-mL slurry containing 10% (w/w) dry solids. Enzyme was dosed on a whole biomass basis because the amount of sample from pretreatment was too small to allow compositional analysis of the sample. The enzyme (Cellic CTec2, Novozymes) loading was 20 mg/g dry biomass, determined using an in-house colorimetric protein assay. This enzyme loading corresponds to 40 to 60 mg protein per gram glucan in the pretreated solids, depending on the composition of the pretreated solids, which was not measured. Enzymatic hydrolysis experiments were performed at 48°C for 5 to 7 days. At the conclusion of each experiment, a liquor sample was removed, filtered, and analyzed for monomeric sugars. The calculated results of the enzymatic hydrolysis experiments were determined from knowledge of the initial dry weight of biomass and the sugar content of the liquor sample. This calculation introduces some bias in the sugar release data, which is discussed elsewhere.

### Data analysis and plotting

Primary analytical data were collected and reduced in Microsoft Excel. Summary data for the two individual assays were further analyzed and plotted using the open source statistical package R [41]. All data and R codes are provided as Additional file 1.

## Abbreviations

CAFI: Consortium for applied fundamentals and innovation; DI: Deionized; FIS: Fraction insoluble solids; HSD: Honestly significant difference; LAP: Laboratory analytical procedure; MVS: Method validation sample; NIR: Near infrared; NREL: National Renewable Energy Laboratory.

## Competing interests

The authors declare that they have no competing interests.

## Authors’ contributions

EW, NN, and CS conceived of and participated in the design of the study. RN performed pretreatment experiments and drafted the relevant section of the manuscript. DP performed saccharification experiments and drafted the relevant section of the manuscript. EW performed data reduction and statistical analysis of all experimental data and drafted the bulk of the manuscript. All authors read and approved the final manuscript.

## Supplementary Material

Additional file 1 Derivation of release and yield values. Click here for file
