# Supplementary material for: A laboratory-scale pretreatment and hydrolysis assay for determination of reactivity in cellulosic biomass feedstocks
Source: Biotechnol Biofuels. 2013 Nov 14;6:162. doi: 10.1186/1754-6834-6-162 (PMC4176505; doi:10.1186/1754-6834-6-162)
Supplement: Additional file 1 — Derivation of release and yield values. [file 1754-6834-6-162-S1.docx]

Derivation of release & yield values

| ***Feedstock Compositional Analysis*** | |  |
| --- | --- | --- |
| *G* | glucan mass fraction in feedstock | -- |
| *X* | xylan mass fraction in feedstock | -- |
| *St* | starch mass fraction in feedstock | -- |
| *Su* | sucrose mass fraction in feedstock | -- |
| ***Experimental Results*** | |  |
| *m_O_* | oven dry weight of biomass before pretreatment PT | g |
| *m_P_* | oven dry weight of biomass sample after PT | g |
| *m_E_* | oven dry weight of biomass sample after enzymatic hydrolysis (EH) | g |
| *g_P_* | glucose released during PT | g |
| *g_E_* | glucose released during EH | g |
| *x_P_* | xylose released during PT | g |
| *x_E_* | xylose release during EH | g |
| ***Conversion Factors*** | | |
| *C_G_* | anhydro MW correction factor for glucose/glucan 180/152 | -- |
| *C_X_* | anhydro MW correction factor for xylose/xylan 150/132 | *--* |

1. ***Pretreatment***

***Sugar Release***

$$R_{PT}^{X}=\frac{x_{P}}{m_{O}} ; R_{PT}^{G}=\frac{g_{P}}{m_{O}}; R_{PT}^{GX}=\frac{g_{P}+x_{P}}{m_{O}}$$

***Sugar Yield (excluding starch & sucrose)***

$$Y_{PT}^{X}=\frac{x_{P}}{m_{O}C_{X}X} ; Y_{PT}^{G}=\frac{g_{P}}{m_{O}C_{G}G}; Y_{PT}^{GX}=\frac{g_{P}+x_{P}}{m_{O}\left( C_{G}G+C_{X}X \right)}$$

***Sugar Yield (including starch & sucrose)***

$$Y_{PT}^{G}=\frac{g_{P}}{m_{O}\left( C_{G}\left( G+St \right)+\frac{Su}{2} \right)}; Y_{PT}^{GX}=\frac{g_{P}+x_{P}}{m_{O}\left( {C_{X}X+C}_{G}\left( G+St \right)+\frac{Su}{2} \right)}$$

1. ***Enzymatic Hydrolysis***

***Sugar Release***

$$R_{EH}^{X}=\frac{x_{E}}{m_{E}} ; R_{EH}^{G}=\frac{g_{E}}{m_{E}}; R_{EH}^{GX}=\frac{g_{E}+x_{E}}{m_{E}}$$

1. ***Combined Pretreatment + Enzymatic Hydrolysis***

***Sugar Release***

$$R_{PT+EH}^{X}=\frac{x_{P}+\frac{m_{P}}{m_{E}}x_{E}}{m_{O}} =R_{PT}^{X}+{\frac{m_{P}}{m_{O}} R}_{EH}^{X}$$

$$R_{PT+EH}^{G}=\frac{g_{P}+\frac{m_{P}}{m_{E}}g_{E}}{m_{O}} =R_{PT}^{G}+{\frac{m_{P}}{m_{O}} R}_{EH}^{G}$$

$$R_{PT+EH}^{GX}=\frac{x_{P}+g_{P}+\frac{m_{P}}{m_{E}}\left( x_{E}+g_{E} \right)}{m_{O}} =R_{PT}^{GX}+{\frac{m_{P}}{m_{O}} R}_{EH}^{GX}$$

***Sugar Yield (excluding starch & sucrose)***

$$Y_{PT+EH}^{X}=\frac{x_{P}+\frac{m_{P}}{m_{E}}x_{E}}{m_{O}C_{X}X}=\frac{1}{C_{X}X}\left( R_{PT}^{X}+{\frac{m_{P}}{m_{O}} R}_{EH}^{X} \right)$$

$$Y_{PT+EH}^{G}=\frac{g_{P}+\frac{m_{P}}{m_{E}}g_{E}}{m_{O}C_{G}G}=\frac{1}{C_{G}G}\left( R_{PT}^{G}+{\frac{m_{P}}{m_{O}} R}_{EH}^{G} \right)$$

$$Y_{PT+EH}^{GX}=\frac{{x_{P}+g}_{P}+\frac{m_{P}}{m_{E}}\left( {x_{E}+g}_{E} \right)}{m_{O}\left( {C_{X}X+C}_{G}G \right)}=\frac{1}{\left( C_{X}X+C_{G}G \right)} \left( R_{PT}^{GX}+{\frac{m_{P}}{m_{O}} R}_{EH}^{GX} \right)$$

***Sugar Yield (including starch & sucrose)***

$$Y_{PT+EH2}^{G}=\frac{g_{P}+\frac{m_{P}}{m_{E}}g_{E}}{m_{O}\left( C_{G}\left( G+St \right)+\frac{Su}{2} \right)}= \frac{1}{\left( C_{G}\left( G+St \right)+\frac{Su}{2} \right)} \left( R_{PT}^{G}+{\frac{m_{P}}{m_{O}} R}_{EH}^{G} \right)$$

$$Y_{PT+EH2}^{GX}=\frac{x_{P}+g_{P}+\frac{m_{P}}{m_{E}}\left( x_{E}+g_{E} \right)}{m_{O}\left( {C_{X}X+C}_{G}\left( G+St \right)+\frac{Su}{2} \right)}= \frac{1}{\left( C_{X}X+C_{G}\left( G+St \right)+\frac{Su}{2} \right)} \left( R_{PT}^{GX}+{\frac{m_{P}}{m_{O}} R}_{EH}^{GX} \right)$$

Note: there is no way to accurately calculate sugar yield from EH alone, with or without starch & sucrose.

1. we do not know the glucan or xylan content of the washed pretreated solids.

2. while we are confident that the sucrose is hydrolyzed during PT, we do not know how much of the starch was hydrolyzed during PT.
